# Estimation of Phase Ratio in Bulk, Textured TWIP/TRIP Steels from Pole Figures

**DOI:** 10.3390/ma14154132

**Published:** 2021-07-24

**Authors:** Marton Benke, Adrienn Hlavacs, Ferenc Kristaly, Mate Sepsi, Valeria Mertinger

**Affiliations:** 1 Institute of Physical Metallurgy, Metalforming and Nanotechnology, University of Miskolc, H-3515 Miskolc-Egyetemvaros, Hungary; femhadri@uni-miskolc.hu (A.H.); femsepsi@uni-miskolc.hu (M.S.); femvali@uni-miskolc.hu (V.M.); 2 Institute of Mineralogy and Geology, University of Miskolc, H-3515 Miskolc-Egyetemvaros, Hungary; askkf@uni-miskolc.hu

**Keywords:** texture, pole figure, phase ratio, steel

## Abstract

The volume fraction of austenite (γ), ε martensite and α′ martensite is of key importance in the research of TWIP/TRIP steels. When mechanical loading is involved, the crystallographic texture also develops, which complicates X-ray diffraction-based phase ratio determination. The problem is more pronounced when only a couple, or only one Bragg-reflection can be measured. A solution for such cases is to determine the ratio of the phases based on the pole distribution function of a selected Bragg-reflection of the present phases. In this manuscript, this method is reconsidered for and applied to non-transmittable bulk specimens for the first time in the reflection mode of XRD pole figure measurements. First, the method was applied to a series of γ–α′ powder mixtures. The results were compared to those obtained by the Rietveld method. Afterwards, the technique was applied to strongly textured, bulk TWIP/TRIP steel specimens which were tensile tested at different temperatures. It was shown that the results of the presented method were close to those of the Rietveld technique in the case of powder mixtures. The results of the tensile-tested steels revealed that the α′ content increases with decreasing test temperatures, and the variation of the α′ ratio correlates very well with the ultimate tensile strength versus the temperature, confirming the contribution of the α′ content to the strength of TWIP/TRIP steels.

## 1. Introduction

Steels showing twinning-induced plasticity (TWIP) and/or transformation-induced plasticity (TRIP) are of great interest because they exhibit the combination of high strength and elongation. Such steels undergo γ→ε (TWIP) and/or γ→α′ (TRIP) phase transformations upon mechanical loading. Meanwhile, a crystallographic texture also forms in the parent and forming phase(s) [1,2,3,4,5,6,7,8,9,10,11,12,13]. Thus, the resulting microstructure contains two or three heavily oriented phases. In general, the relative number of phases—or the phase ratio, in short—and the developed crystallographic texture are both of interest. Electron backscattered diffraction (EBSD) is an effective technique to provide information about both [3,4,5,7,9,11,12]. This method is useful to examine the relationships of neighbouring crystals, such as misorientation. However, its drawback is that the examined volume is rather small, and an overall description can be obtained through the examinations of multiple fields of inspection. X-ray diffraction (XRD) provides more comprehensive information about the texture compared to EBSD. Usually, the intensity functions detected during the texture measurements are solely used to characterize the crystallographic anisotropy [6,10,13]. The determination of the phase ratio by means of XRD is generally associated with the Rietveld method, which was originally developed for powders and samples with only a weak texture [14,15]. Although this method provides accurate data for texture-free cases and samples with a relatively weak texture, its accuracy is questionable if the crystallographic anisotropy is strong. The problem was known from the earliest techniques. Alexander et al. showed that the volume fraction of a crystalline component can be calculated from the integral intensities of all of the Bragg reflections, or from a set of Bragg reflections [16]. If a texture is present, however, the measured intensities of the reflections are disturbed. In general, there are two ways to deal with the effect of texture. One possibility is to calculate the texture factors which characterize the intensity change caused by the texture. In the earlier works, texture factors were determined by dividing the measured intensities by the texture-free intensities of all of the measurable {hkl} reflections obtained from one direction [17,18]. The first version was applicable for relatively low degrees of preferred orientation, which was refined for stronger textures by Dickson [19]. He also highlighted that it could cause a high error if the preferred orientation results in a non-measurable Bragg reflection in the direction of the examination. The modified method was still based on measured and close-to-random intensities of a set of Bragg reflections from only one direction. According to Bunge et al., the texture factors of each {hkl} reflections can be determined from the coefficients of the series expansion. This method requires a sufficient number of experimental intensities of some {hkl} reflections from more than one direction [20]. To summarize, the principle of texture factor calculation methods is to determine the deviation of the normalized intensities of the measured {hkl} reflections from the texture-free intensities. Therefore, in order to apply these methods, a set of Bragg angles needs to be measured. There are some cases, however, when—due to strong texture—only a few (or only one) Bragg reflections appear on the XRD spectrum from a given phase. This often occurs in TWIP/TRIP steels; thus, the previous methods fail in such cases. This problem can be handled by eliminating the effect of texture. Wasserman and Grewen showed first that this can be achieved through the measurement of the complete pole distribution function (PDF, e.g., the pole figure) of the Bragg reflection, as in Equation (1) [20,21,22]:

(1) $$I_{\left\{hkl\right\}}^{Random}=\frac{1}{4\pi }\int_{\alpha =0}^{\pi }\int_{\beta =0}^{2\pi }I_{\left\{hkl\right\}}\left(\alpha \beta \right)\sin\alpha d\alpha d\beta$$

where $I_{\left\{hkl\right\}}^{Random}$ is the texture-corrected intensity which would be measured if the sample was texture-free, *α* is the angle of sample tilting, *β* is the angle of sample rotation, and $I_{\left\{hkl\right\}}\left(\alpha \beta \right)$ is the measured intensity in a given (*αβ*) direction [20,21,22]. In the case of fibre textures, the pole distribution is rotationally symmetrical; therefore, Equation (1) can be rewritten as Equation (2) [20,22]:

(2) $$I_{\left\{hkl\right\}}^{Random}=\frac{1}{2}\int_{\alpha =0}^{\pi }I_{\left\{hkl\right\}}\left(\alpha \right)\sin\alpha d\alpha$$

In order to obtain complete pole figures, texture measurements must be carried out in the transmission mode. Brokmeier successfully used this method to determine the phase ratios in Al-Cu composites and graphite–silicon carbides, both with a strong fiber texture [22]. He used the texture-corrected random intensity as the input data for the phase ratio calculation proposed by Klug and Alexander [16]. His calculations were based on transmission neutron diffraction measurements performed on transmittable samples. It is worth mentioning that the Rietveld method can also be applied to the results of neutron diffraction [23]. Although it was shown that phase ratios can be determined reliably based on full-pole distributions, the drawback of this method is that it can only be applied to transmittable samples in the transmission mode, and not the more frequently used reflection mode. The further disadvantage of neutron diffraction is that it requires a neutron source, which is quite rare, whereas laboratory X-ray diffractometers are a widely used piece of equipment.

The objective of this manuscript was to reconsider the phase ratio calculation method proposed by Wassermann and Grewen, and to apply it to strongly textured, bulk steel specimens showing TWIP/TRIP behaviour measured in the reflection mode of XRD texture examinations. The phase ratios were calculated using both Wassermann and Grewen’s formulae, and a simplified, reconsidered formula. The results are discussed with respect to the measured ultimate tensile strength (UTS) values.

## 2. Materials and Methods

**Figure 1:**
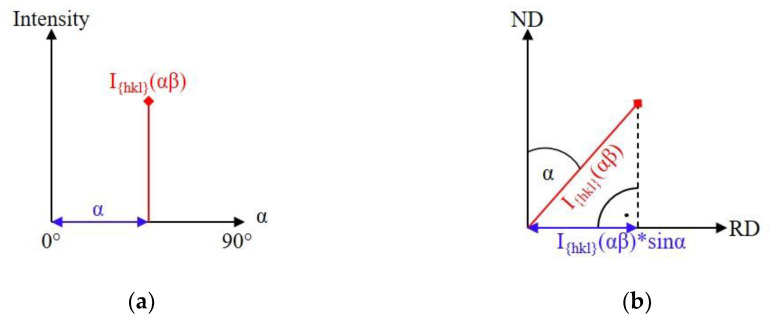
Intensity measured at the tilting angle α at rotation angle β (**a**) in the pole-figure coordinate system, and (**b**) in the sample coordinate system together with its vertical projection on the rolling plane. RD: rolling direction, ND: normal direction of the sample [24] Reprinted with permission from Marton Benke (2020) Crystallography Reports.

In this work, instead of calculating the texture-free (random) intensity of a Bragg reflection, the sum intensity of the {hkl} pole figure is determined using Equation (3).

(3) $$I_{\left\{hkl\right\}}^{PF*}=\int_{\alpha =0}^{\frac{\pi }{2}}\int_{\beta =0}^{2\pi }I_{\left\{hkl\right\}}\left(\alpha \beta \right)d\alpha d\beta$$

where $I_{\left\{hkl\right\}}^{PF*}$ is the sum intensity of the *{hkl}* pole figure, α is the angle of the sample tilting, β is the angle of the sample rotation, and *I_{hkl}_*(*αβ*) is the measured absorption (defocus)-corrected intensity of the *{hkl}* reflection in a given (*αβ*) direction. Note that there are two relevant differences between Equations (1) and (3). First, the divider “2*π*” of Equation (1) is neglected in order to obtain the full intensity of the pole figure instead of the texture-free intensity. Second, the multiplier term “sinα” of Equation (1) is omitted. This is because weighting *I_{hkl}_*(*αβ*) with sinα gives the vertical projection of *I_{hkl}_*(*αβ*) on the rolling plane, which has no physical sense in the calculation of the total intensity of a pole figure (Figure 1) [24].

Nevertheless, a comparison between the results obtained from the examined samples using Equations (1) and (3) will be given. The integrations in Equation (3) can be performed physically. This means that *I_{hkl}_* can be measured in a set of α tilting positions and β rotations. Thus, in practice, Equation (3) can be rewritten as:

(4) $$I_{\left\{hkl\right\}}^{PF*}=\overset{\alpha_{Max}}{\underset{\alpha =0}{\sum }}\sum_{\beta =0}^{2\pi }I_{\left\{hkl\right\}}\left(\alpha \beta \right)$$

where α*_Max_* is the upper limit of the sample tilting that can be realized with the equipment. The phase ratio calculations were based on Equation (5) [25]:

(5) $$\frac{I_{X,\left\{hkl\right\}}}{I_{Y,\left\{hkl\right\}}}=\frac{K_{p,X,\left\{hkl\right\}}V_{X}}{K_{p,Y,\;\left\{hkl\right\}}V_{Y}}$$

where *I_X,{hkl}_* is the intensity of the *{hkl}* reflection of phase *X*, *K_p,X,{hkl}_* is the phase-related factor of the *{hkl}* reflection of phase *X,* and *V_X_* is the relative amount of phase *X* [25]. From this, Equation (6) was derived, where $I_{X,\left\{hkl\right\}}^{PF*}$_}_, the sum intensity of the *{hkl}* pole figure of phase *X,* was used instead of the intensity *I_X,{hkl}_*.

(6) $$V_{X}:V_{Y}=\frac{I_{X,\left\{hkl\right\}}^{PF*}}{K_{p,X,\left\{hkl\right\}}}:\frac{I_{Y,\left\{hkl\right\}}^{PF*}}{K_{p,Y,\left\{hkl\right\}}}$$

For the three-phase steel samples, Equation (7) was used.

(7) $$V_{X}:V_{Y}:V_{Z}=\frac{I_{X,\left\{hkl\right\}}^{PF*}}{K_{p,X,\left\{hkl\right\}}}:\frac{I_{Y,\left\{hkl\right\}}^{PF*}}{K_{p,Y,\left\{hkl\right\}}}:\frac{I_{Z,\left\{hkl\right\}}^{PF}}{K_{p,Z,\left\{hkl\right\}}}$$

No absorption coefficient was used here, because the materials to be tested consisted of austenite, α′ martensite and ε martensite, of which the absorption coefficients are considered to be equal.

Powder samples were prepared with different γ/α′ phase ratios for the validation measurements. The particle size was around 90 µm for the austenite and around 60 µm for the α′ powders. The required quantities of the two powders were mixed in a glass jar by hand. The mixtures were first placed in a resin mount, and the resin was poured onto them with continuous stirring. After fixing, flat discs were machined from the resin-mounted mixtures, which were used for the XRD examinations. Three steel types with different Cr contents were prepared by TU Bergakademie Freiberg Freiberg, Germany. The compositions of the examined steels are summarized in Table 1.

Tensile test specimens with diameters of 5 mm were machined from the as-received hot-rolled rods. The test specimens were subjected to a solution heat treatment at 1273 K (1000 °C) in an argon atmosphere for 30 min, followed by water quenching to room temperature. Afterwards, tensile tests were performed until fracture at different test temperatures. The test equipment was an Instron 5982 universal mechanical tester equipped with a specimen furnace (Instron, Norwood, USA). Before loading, the specimens were heated to 573 K (300 °C) to obtain a single-phase austenitic structure, and were held at this temperature for 30 min. After that, the specimens were cooled down to the tensile test temperatures, which were chosen to be 473 K (200 °C), 453 K (180 °C), 433 K (160 °C), 413 K (140 °C), 398 K (125 °C), 383 K (110 °C) and room temperature. Tensile tests were carried out with a 3 mm/min strain rate. Cross sections were prepared from the fractured test specimens for the texture examinations [13]. The Rietveld analysis was performed using a Bruker D8 Discover diffractometer with a DaVinci control, equipped with energy-sensitive LynxEYE XE-T line detectors, a CuKα X-ray source and the equipment’s Topas V 4.0 software. The XRD texture measurements were carried out with a Bruker D8 Advance X-ray diffractometer equipped with a Eulerian cradle and a CoKα anode (Bruker, Billerica, USA). Incomplete pole figures in the α = 0…75° tilting range were recorded. Thus, the complete pole distribution functions were not used, but rather incomplete pole figures in the α = 0…75° tilting range. In the case of two-phase powder samples, the γ{111} and α′{110} pole figures were measured, while for TWIP/TRIP steels, the ε{002} pole figure was measured in addition. The pole figures were evaluated with Texeval, the software of the device.

## 3. Results

### 3.1. Two-Phase Powder Mixtures

**Figure 2:**
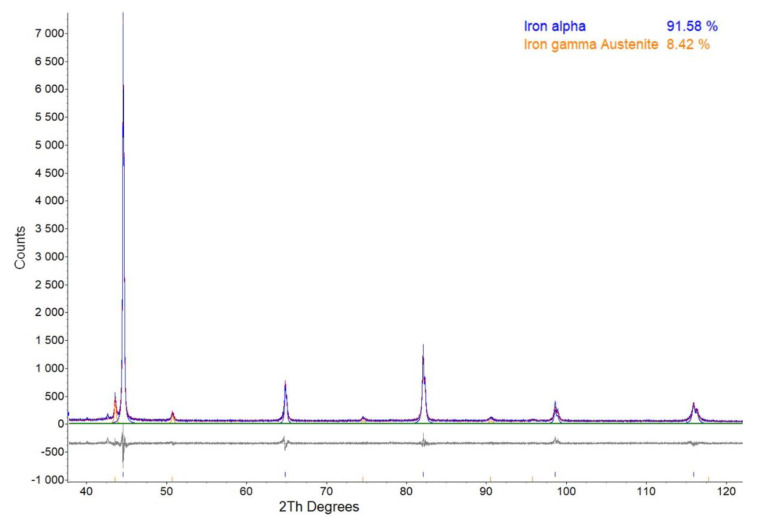
Measured full-spectrum, fitted interference function and difference curve of a powder mixture.

**Figure 3:**
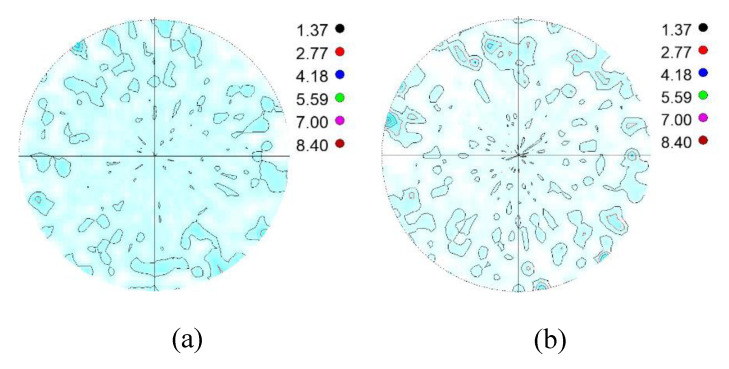
(**a**) α′{110} and (**b**) γ{111} pole figures of the powder mixture containing 72.6% α′ (according to the Rietveld method).

Figure 2 shows an example for the measured XRD spectrum, the fitted full interference function and the difference curve used for the Rietveld analysis of the powder mixture. Figure 3 shows the measured, absorption corrected α′{110} and γ{111} pole figures of a powder mixture specimen. The equi-density levels are marked with coloured lines, and their levels are given next to the pole figures. In addition, the saturation of the pole figures is proportional to the pole density.

**Figure 4:**
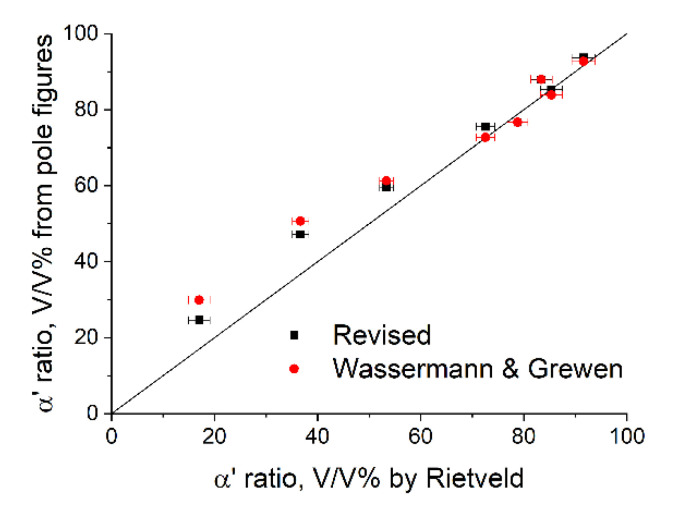
The ratio of α′ martensite in the prepared α′–γ powder mixtures determined with the Rietveld method and calculated from the pole figures using Wassermann and Grewen’s formula and the revised formula.

The relative amount of α′ martensite in the prepared powder mixtures determined by the Rietveld method and calculated from the pole distribution functions according to Wassermann and Grewen’s formula and the revised formula are summarized in Table 2. The error of the Rietveld method is included. The differences compared to the results of the Rietveld method (Δ) are also given. The results are also plotted in Figure 4, in which the solid line represents the equivalency between the Rietveld method and pole distribution-based methods.

It can be seen that the results obtained with the refined formula are generally closer to those of the Rietveld method than the results using Wassermann and Grewen’s formula. In the case of Wassermann and Grewen’s formula, the largest deviation from the results of the Rietveld method is 14.0 V/V% (50.7 V/V% instead of 36.7 V/V%), while in the case of the revised formula, it is 10.5 V/V% (47.2 V/V% instead of 36.7 V/V%). Apart from this sample, the value of Δ is below 10 using the refined formula. Based on the results obtained for the powder mixtures, it can be stated that the pole-distribution function-based methods provide a good estimation of the phase ratios. It can also be deduced that the revised formula gives a better accuracy compared to the formula originally proposed by Wassermann and Grewen.

### 3.2. Bulk Tensile Tested TWIP/TRIP Steels

**Figure 5:**
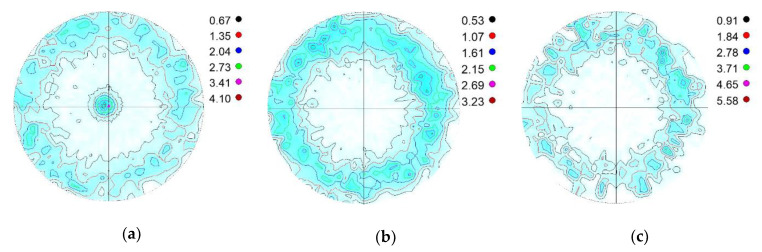
(**a**) γ{111}, (**b**) ε{002} and (**c**) α′{110} pole figures of Steel 0.07, tensile tested at 473 K [13].

Figure 5 shows the absorption-corrected γ{111}, ε{002} and α′{110} pole figures of Steel 0.07 tensile tested at 473 K. These pole figures were chosen because they represent the habit planes of the martensitic γ→ε and γ→α′ transformations, and are high-intensity reflections. The tensile direction is perpendicular to the projection plane; thus, it is in the center of the pole figures.

**Figure 6:**
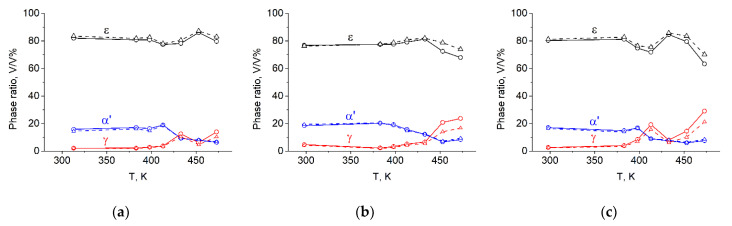
Phase ratios calculated from the measured, absorption-corrected γ{111}, α′{110} and ε{002} pole figures of the steel specimens tensile tested at different temperatures. The results obtained with the method proposed by Wassermann and Grewen are marked with dashed lines; those obtained with the revised method are marked with solid lines. (**a**) Steel 0.07, (**b**) Steel 2.26, (**c**) Steel 6.12.

As seen in the figure, a strong fibre texture formed in all three phases of Steel 0.07 tensile tested at 473 K. In nature, similar textures developed in all of the specimens. All of the pole figures are presented, and the developed textures are discussed in detail in [13]. The phase ratios of austenite, α′ martensite and ε martensite as a function of the tensile test temperature are shown in Figure 6. The results obtained with the method proposed by Wassermann and Grewen are marked with dashed lines, while those obtained with the revised method are marked with solid lines.

**Figure 7:**
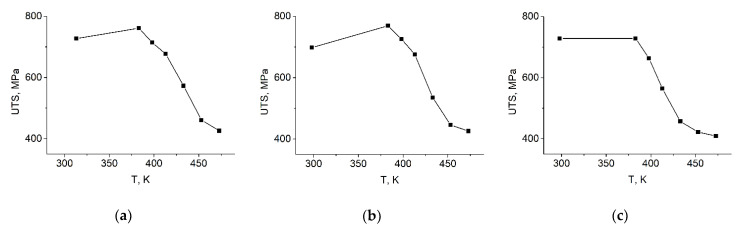
Ultimate tensile strength values of the steel specimens tensile tested at different temperatures. (**a**) Steel 0.07, (**b**) Steel 2.26, (**c**) Steel 6.12.

It can be seen that the determined phase ratios vary with the test temperature. Besides some fluctuations, it is evident that the amount of austenite is larger at higher temperatures and smaller at lower temperatures, regardless of the Cr content. In accordance with this, the amount of α′ martensite is smaller (~8 V/V%) at higher temperatures, while it is notably larger (~20 V/V%) at lower temperatures. The amount of ε martensite is the largest in every specimen, being ~80 V/V%. Beside some fluctuations, a tendency in the ε martensite content versus the temperature cannot be observed. If one compares the results calculated according to what Wassermann and Grewen originally proposed and the revised method, it can be stated that there are some differences in the phase ratio values, but the trends of the phase variations versus the temperature are the same. Thus, similar dependencies of the phase content on the temperature can be observed using both methods. Figure 7 shows the UTS values of the examined steels as a function of the tensile test temperature.

In all three examined steels, the UTS values were between 700 and 800 MPa at the lower test temperatures, and they decreased to ~400 MPa at higher temperatures. Comparing this tendency to the α′ martensite phase ratio shown in Figure 6, it can be observed that the amount of α′ martensite and the UTS values correlate very well.

## 4. Discussion

It was seen that in the case of texture-free α′–γ powder mixtures, the results calculated from the full pole density functions slightly deviated from the results of the Rietveld analysis, which could have two probable sources. First, the CoKα source was used for the texture measurements, while CuKα was used during the Rietveld analysis. The penetration depths (at 90% intensity) for the two wavelengths are notably different, namely 11.4 μm and 2.0 μm, respectively. Although the α′ and γ powders were mixed before embedding, a slight heterogeneity in depth could be present within the embedded mixtures, because particles could have been removed from the surface during the mechanical polishing. This cannot be completely excluded, even with the most careful sample preparation. Based on previous practice, it was found that γ particles are more prone to be removed from the resin. The particle removal appears only on the surface, but this region represents a different fraction of the examined volume for different penetration depths. Thus, slightly different α′/γ ratios could be within the examined volumes of the two diffractometers. Because the samples with a lower α′ ratio have more γ particles, more γ particle removal is expected during sample preparation. This is the reason why the difference between the results of the Rietveld and pole figure-based methods is larger at lower α′ ratios. Second, the sample tilting could be performed only up to αMAX = 75° during the pole figure measurements. Consequently, incomplete pole distribution functions were recorded and used. Theoretically, the pole density function is homogeneous along α in the case of powders. However, if texture is present within the examined sample, different fractions of the full intensity can be present above αMAX—and could be unmeasured—for the different phases. Thus, using incomplete pole distribution functions instead of complete ones can decrease the accuracy of the measurements. Note that this did not notably affect the results of the tensile-tested specimens, as will be explained later. If better accuracy is required, the presented method can be developed with the use of the complete pole figures obtained using the transmission mode. It was also shown that the results obtained using the revised formula instead of Wassermann and Grewen’s yielded closer results to those of the Rietveld method. Because the Rietveld method is generally accepted as one of the most accurate methods to determine the phase ratios of powder mixtures, it can be stated that the revised method gives more accurate results than the formula proposed by Wassermann and Grewen. The largest deviation between the results of the Rietveld method and the revised one was that pole density function-based method was found to be ~10 V/V%. In the case of powders, this error is notable considering the accuracy of the Rietveld method. However, in the case of heavily textured samples, the applicability of the Rietveld method is questionable, but the proposed method can still be used with the same accuracy. In the case of strongly textured, multiphase steels, the Rietveld method was not used. As was mentioned earlier, texture measurements were performed in the reflection mode during which the sample tilting was realized up to αMAX = 75°. Due to the developed fiber textures, the vast majority of the poles were found to be within the examined α range for all three phases. Nevertheless, because some intensity could appear above the realized αMAX = 75° tilting, the presented technique can be considered as an estimation method for the phase ratios. It is worth mentioning that in the presented case, the tensile tested samples were so heavily textured that only the strongest examined Bragg reflections could be measured. However, if a series of reflections can be measured, the presented method can be further developed in such a way that the pole figures of a series of reflections can be included in the phase ratio calculation. The mentioned developments, however, exceed the length of a research paper and require further research. Finally, a strong correlation was observed between the variation of the α′ ratio and the measured UTS as a function of temperature. This confirms that the quantity of α′ martensite has a notable contribution to the strength of steels undergoing the γ→α′ (γ→ε→α′) martensitic transformation during mechanical loading. It was also seen that in case of Steel 2.26, the UTS and α′ ratio have maximums at the same test temperature, whereas for the other two steels, their maximum values slightly differed (up to 30 K). The explanation of this is that the UTS values are determined by the behavior of the full length of the gauge section of the tensile test specimens. However, phase ratios were determined on cross sectional samples taken from one specific location along the gauge length. This shows that the phase ratio of TWIP/TRIP steels is similar, but not necessarily exactly the same along the full length of the gauge section. Based on this and the observed variation of the phase ratio vs the temperature, it can be deduced that the α′ content of the total gauge section is the highest at that tensile test temperature, where the UTS is the highest; however, slight differences in α′ content can be present along the gauge section of the tensile test specimens.

## 5. Conclusions

The phase ratio estimation method based on the incomplete pole distribution function of one Bragg reflection of each phase was presented. The method was validated on texture-free powder samples and applied to three steels undergoing both TWIP and TRIP effects. Based on the obtained results, the following conclusions could be deduced:

(1) The phase ratio determination method proposed by Wassermann and Grewen can be simplified by determining the intensities of the pole figures instead of calculating the texture-free (random) intensities, and by omitting the “sinα” multiplier term, which has no reasonable role in the calculation.
(2) The revised method can be realized on non-transmittable, bulk steel samples through X-ray diffraction pole figure measurements performed in the reflection mode up to 75° tilting.
(3) In the case of texture-free powder samples, the results of the revised method were in good agreement with the results of the Rietveld refinement method.
(4) In the case of steels showing both TWIP and TRIP behaviour, it was found that at lower tensile test temperatures, the relative amount of α′ martensite is larger than at higher test temperatures, while the amount of austenite varies inversely. The UTS values correlate well with the relative amount of α′ martensite, which is linked to the strengthening effect of α′ martensite.
(5) It was concluded that in cases when every pole is within the realized tilting range, the revised method can be applied in the reflection mode of XRD pole-figure measurements to estimate the phase ratios of bulk specimens.

## Figures and Tables

**Table 1:** Chemical compositions of the examined steels [13].

Steel Type	Composition, wt%				
	C	Mn	Cr	Si	S
Steel 0.07	0.03	18.00	0.07	0.03	0.03
Steel 2.26	0.03	17.70	2.26	0.10	0.03
Steel 6.12	0.08	17.70	6.12	0.06	0.03

**Table 2:** The relative amount of α′ martensite in the prepared mixtures, determined by the Rietveld method, Wassermann and Grewen’s formula, and the revised formula, V/V%.

		α′ Content, V/V%			
Rietveld		Waasermann & Grewen		Revised	
Measured	Error	Calculated	Δ	Calculated	Δ
17.0	4.15	29.9	12.9	24.6	7.6
36.7	3.17	50.7	14.0	47.2	10.5
53.4	2.67	61.3	7.9	59.6	6.2
72.6	3.63	72.7	0.1	75.5	2.98
78.8	3.94	76.7	2.1	76.7	2.1
83.4	4.17	88.0	4.6	87.9	4.5
85.4	4.27	83.9	1.5	85.3	0.1
91.6	4.58	92.7	1.1	93.7	2.1

## Data Availability

The data presented in this study are available on request from the corresponding author.
